# Combined Low-Dose Isotretinoin and Sequential Multimodal Laser Therapy for Active Acne and Erythematous Atrophic Scars: A Five-Patient Case Series

**DOI:** 10.7759/cureus.109680

**Published:** 2026-05-26

**Authors:** Pablo Pagano, Agustina B Medina, Pablo Russo, Alessandra Zevini, Daniela Martinelli, Riccardo Barini

**Affiliations:** 1 Dermatology, Dermis Dermoestética, Neuquén, ARG; 2 Dermatology, Universidad Abierta Interamericana, Buenos Aires, ARG; 3 Clinical and Medical Affairs, El.En. Group, Calenzano, ITA

**Keywords:** 1064 nm nd:yag laser, 1540 nm er:glass laser, acne scars, acne vulgaris, fractional co₂ laser, isotretinoin, sequential laser treatment

## Abstract

The simultaneous presence of active inflammatory acne and atrophic acne scarring represents a challenging clinical scenario. Although isotretinoin remains the gold standard for severe acne, its combined use with laser procedures has historically been restricted due to safety concerns regarding wound healing. This study aimed to evaluate the safety and clinical efficacy of low‑dose systemic isotretinoin combined with sequential multimodal laser therapy for the concurrent treatment of active acne and atrophic acne scars. This retrospective case series included five patients with severe inflammatory acne and atrophic scarring treated with fixed low‑dose isotretinoin (20 mg/day) alongside a sequential multimodal laser protocol, based on non‑ablative fractional 1,540 nm laser, with fractional CO₂ and 1,064 nm Nd:YAG lasers introduced selectively and sequentially according to clinical criteria. Acne severity was assessed using the Investigator’s Global Assessment (IGA), while scar severity was evaluated using the Echelle D'Evaluation Clinique des Cicatrices D'Acne (ECCA) score and the Goodman & Baron quantitative scale. Outcomes and adverse events were recorded over a three‑month follow‑up. All patients achieved near‑complete or complete acne clearance (IGA 0-1) within one month of treatment initiation, with results maintained at the three‑month follow‑up. Clinically meaningful improvements in scar severity were observed, with mean reductions of 87.9% in ECCA scores and 84.4% in Goodman & Baron scores. No serious adverse events or impaired wound healing were reported. This case series suggests that low‑dose isotretinoin can be safely combined with sequential multimodal laser therapy to simultaneously address active inflammatory acne and severe atrophic scarring. Larger prospective studies are warranted to confirm these findings and optimize treatment protocols.

## Introduction

Acne vulgaris is a chronic inflammatory disorder with a multifactorial pathogenesis, affecting up to 85% of adolescents and persisting into adulthood in approximately one‑fifth of cases [1]. Although inflammatory lesions may resolve, acne frequently leads to permanent atrophic scarring, particularly in severe forms of the disease, with reported rates of up to 82% [2]. These scars result from altered wound‑healing processes following follicular rupture and dermal inflammation, characterized by excessive collagen degradation and irreversible dermal volume loss [3].

The coexistence of active inflammatory lesions and established atrophic scarring represents a particularly challenging clinical scenario. In these patients, ongoing follicular inflammation exacerbates pre-existing dermal damage, creating a structurally compromised tissue environment where each new inflammatory episode risks further scar formation. This dual pathology, acute inflammation superimposed on chronic structural defects, necessitates integrated therapeutic strategies capable of simultaneously suppressing active disease while addressing established scarring.

Systemic isotretinoin remains the cornerstone of therapy for moderate-to-severe acne, providing prolonged remission by targeting multiple pathogenic pathways, including sebaceous gland hyperactivity, follicular hyperkeratinization, and inflammatory mediators. However, its historical association with impaired wound healing and concerns about procedural safety have long restricted its combined use with laser‑based treatments. Early reports from the 1980s and 1990s describing hypertrophic scarring, delayed re‑epithelialization after dermabrasion, and keloid formation following vascular laser treatments performed during isotretinoin therapy led to the long‑standing recommendation to postpone such interventions for at least six months after isotretinoin discontinuation [4-7]. For decades, this caution limited the integration of laser technologies in patients who might benefit most from early intervention, particularly those with active acne and evolving scarring.

This paradigm has been progressively reevaluated in the past decade, as emerging evidence has challenged earlier assumptions regarding procedural safety. Multiple retrospective and prospective studies have reported normal wound healing, absence of hypertrophic or keloid scarring, and favorable tolerability profiles when modern fractional and non‑ablative laser technologies are performed during or shortly after low‑dose isotretinoin therapy [8,9-11]. These findings are further supported by expert consensus statements and task force recommendations, which have concluded that the available evidence does not justify routinely delaying procedures such as non‑ablative lasers, fractional resurfacing, or superficial ablative treatments in patients receiving isotretinoin [12].

These evolving safety data may allow for earlier consideration of integrated treatment strategies in selected patients, particularly those with coexisting active inflammation and progressive scarring. The rationale for such early intervention lies in the complementary biological mechanisms of current laser systems, which can be used either individually or in combination to optimize tissue remodeling. Modern laser platforms provide non‑overlapping effects, including non‑ablative dermal coagulation (1,540 nm) [13], micro‑ablative remodeling of fibrotic tissue (fractional CO₂) [14], and vascular or photoacoustic modulation (1,064 nm) [15,16], together offering a strong rationale for early, integrated laser‑based intervention. Clinical evidence supports the efficacy of dual‑wavelength regimens, with CO₂ + Er:Glass demonstrating benefits in atrophic acne scars and CO₂ + 1,064 nm showing therapeutic value across multiple scar types, including immature hypertrophic scars, striae distensae, and atrophic scarring [17-22]. However, no studies have evaluated sequential protocols combining all three wavelengths, nor has this triple-combination approach been examined specifically in patients with acne undergoing concomitant isotretinoin treatment. Despite emerging data supporting isotretinoin safety with fractional non-ablative lasers, evidence regarding concurrent use with ablative fractional CO₂ resurfacing, historically considered high-risk due to concerns of impaired re-epithelialization, remains limited.

The present study aims to evaluate the safety, tolerability, and clinical outcomes of combining low‑dose systemic isotretinoin with sequential multimodal laser therapy, including 1,540 nm non‑ablative fractional, fractional CO₂, and 1,064 nm Nd:YAG lasers, in patients presenting with active inflammatory acne and severe atrophic scarring.

## Case presentation

This retrospective, single-center case series was conducted in a private dermatology practice and included five patients presenting with long‑standing mild-to-severe inflammatory acne and acne scars, all of whom had previously failed standard topical and/or systemic therapies. Inclusion criteria required provision of informed consent and absence of systemic antibiotic therapy within the preceding eight weeks, as well as topical acne therapies in the two weeks preceding initiation of the combined protocol. Patients with a history of keloids, abnormal wound healing, recent dermal fillers, or prior facial resurfacing procedures within three months were excluded. Pregnant or breastfeeding individuals were also not considered for treatment. All patients received isotretinoin at a daily dose of 20 mg throughout the entire study period.

The laser protocol consisted of six monthly sessions for all patients, conducted within a structured sequential multimodal framework, with laser modalities applied selectively and sequenced according to predefined clinical criteria, as detailed in Table 1. It combined wavelengths with different targets: the 1,540 nm Er:Glass laser for dermal remodeling, fractional CO₂ for the treatment of atrophic scars, and the 1,064 nm Nd:YAG laser for sebaceous and vascular modulation.

**Table 1:** Sequential multimodal laser treatment specifications. Detailed breakdown of the laser modalities employed per session for each patient. The protocol utilized a combination of 1,540 nm and fractional CO₂ from the initial sessions, with the progressive incorporation of 1,064 nm Nd:YAG laser. Nd:YAG treatments were delivered in Q‑switched mode (QS) and/or quasi–long‑pulsed mode (P), depending on clinical indication.

Patient	Session 1	Session 2	Session 3	Session 4	Session 5	Session 6
1	1,540 + CO₂	1,540 + CO₂	1,540 + CO₂ + Nd:YAG P and QS	1,540 + CO₂ + Nd:YAG P and QS	1,540 + CO₂ + Nd:YAG P and QS	1,540 + CO₂ + Nd:YAG P and QS
2	1,540 + CO₂	1,540 + CO₂ + Nd:YAG QS	1,540 + CO₂ + Nd:YAG QS	1,540 + CO₂ + Nd:YAG QS	1,540 + CO₂ + Nd:YAG QS	1,540 + CO₂ + Nd:YAG QS
3	1,540 + CO₂	1,540 + CO₂	1,540 + CO₂	1,540 + CO₂ + Nd:YAG P and QS	1,540 + CO₂ + Nd:YAG P and QS	1,540 + CO₂ + Nd:YAG P and QS
4	1,540 + CO₂	1,540 + CO₂	1,540 + CO₂ + Nd:YAG QS	1,540 + CO₂ + Nd:YAG QS	1,540 + CO₂ + Nd:YAG QS	1,540 + CO₂ + Nd:YAG QS
5	1,540	1,540	1,540 + Nd:YAG QS	1,540 + Nd:YAG QS	1,540 + Nd:YAG QS	1,540 + Nd:YAG QS

During the first sessions, patients were treated using a mixed 1,540 nm/CO₂ fractional laser (Youlaser MT, Quanta System). For the 1,540 nm non‑ablative fractional laser, patients received 7-8 W per shot, with a pulse width of 4-5 ms, using a 100‑dot pattern (28-40 mJ per dot), one stack, and two passes per session. For the fractional CO₂ component, treatments were performed using a 100‑dot pattern with a pulse width of 0.5 ms. Power settings ranged from 9 to 16 W, resulting in an energy delivery of approximately 4.5-8 mJ per dot. Depending on the clinical response and scar characteristics, one or two stacks were applied per spot.

Based on the protocol‑defined clinical criteria, including acne activity, erythematous/vascular component, and scar evolution, a 1,064 nm Nd:YAG laser (Chrome, Quanta System) was progressively incorporated into the treatment protocol at varying timepoints across the patient cohort. In patients with a marked inflammatory and vascular component, the Nd:YAG laser was incorporated at earlier stages to target inflammatory and vascular pathways, whereas in other cases it was introduced in later sessions when the therapeutic focus shifted toward post‑inflammatory erythema reduction and dermal remodeling. During sessions incorporating multiple modalities, laser treatments were delivered sequentially within the same visit. The Nd:YAG component was administered in quasi-long-pulsed mode (0.3 ms pulse duration, 4 mm spot size, 12-15 J/cm², 7.5 Hz repetition rate) and/or Q-switched mode (6 ns pulse duration, 8 mm fractional spot, 0.5-1 J/cm², 10 Hz), delivered in two passes.

A compounded anesthetic cream (pharmacy-prepared) was applied before each treatment session to ensure adequate local anesthesia. Immediately post-treatment, ice packs were applied for 5-10 minutes to minimize discomfort and erythema. Throughout the study period, patients were instructed to avoid topical retinoids.

Acne severity was assessed using the Investigator’s Global Assessment (IGA), providing a standardized grading of inflammatory and non‑inflammatory lesion burden. Acne scarring was documented using both the Goodman & Baron quantitative scale and the ECCA score, enabling structured evaluation of early scar morphology and its progression over time. Clinical outcomes, supported by photographic documentation, were evaluated at baseline, at each treatment session, and at the follow-up visits. All clinical photographs were obtained under standardized conditions, including consistent patient positioning, camera distance, and lighting across visits, to ensure comparability over time.

Safety and tolerability were monitored in detail throughout the treatment course. At every visit, patients were evaluated for potential adverse events, including post-inflammatory erythema, pigmentary alterations, blistering, prolonged swelling, or acne flares. Treatment pain was recorded using a Visual Analog Scale (VAS), while patient‑reported quality of life was assessed using the Dermatology Life Quality Index at baseline and at the final follow-up. Patients rated their satisfaction with the combined protocol using a five‑point Likert scale, ranging from 1 (extremely satisfied) to 5 (extremely unsatisfied). Individual patient demographics and Fitzpatrick skin types are reported in Table 2.

**Table 2:** Patient characteristics and clinical outcomes. Summary of demographic data, Fitzpatrick skin types, and clinical severity scores (IGA, Goodman & Baron, and ECCA) recorded at baseline, one-month, and three-month follow-up intervals. The data highlights a consistent and clinically meaningful reduction in scar severity across the entire cohort. IGA = Investigator’s Global Assessment; ECCA = Echelle D'Evaluation Clinique des Cicatrices D'Acne; FU = follow-up

Patient	Age (years)	Gender	Fitzpatrick skin type	IGA			Goodman & Baron			ECCA		
				Baseline	1-Month FU	3-Month FU	Baseline	1-Month FU	3-Month FU	Baseline	1-Month FU	3-Month FU
1	20	M	II	5	1	1	31	10	16	325	95	65
2	26	F	III	4	0	0	29	9	0	265	90	60
3	22	M	II	5	1	1	34	11	9	340	60	60
4	12	F	III	4	0	0	27	0	0	295	0	0
5	29	F	III	4	0	0	31	1	0	225	0	0

**Figure 1:**
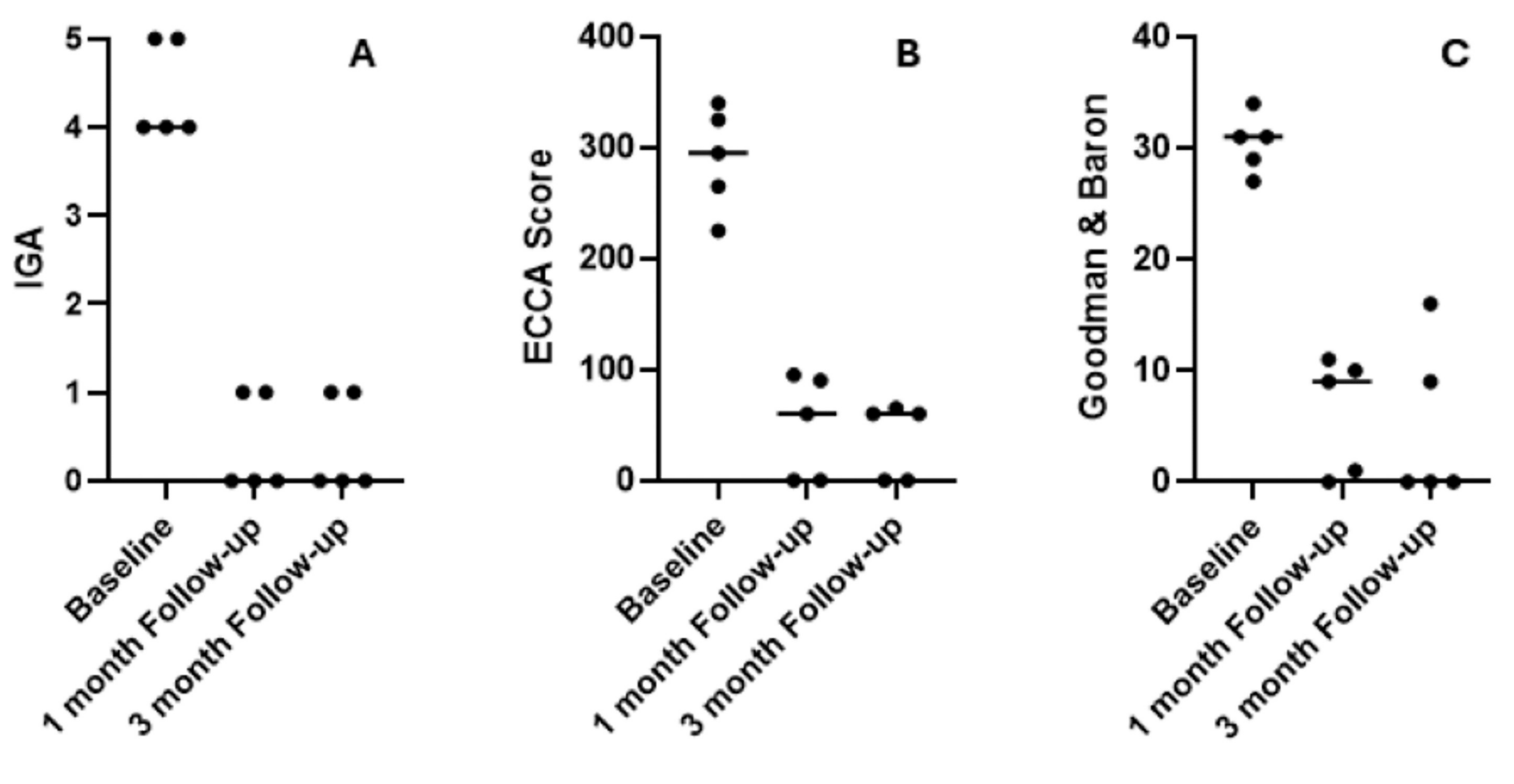
Changes in acne severity and atrophic scar scores over time. Individual patient values (dots) and medians (horizontal lines) for (A) IGA, (B) ECCA, and (C) Goodman & Baron quantitative scale at baseline, one‑month follow‑up, and three‑month follow‑up are shown. A marked reduction in acne and scar severity was observed at one month, with sustained improvement at three months across all scoring systems. IGA = Investigator’s Global Assessment; ECCA = Echelle D'Evaluation Clinique des Cicatrices D'Acne

Treatment with the combined isotretinoin and sequential laser protocol resulted in clinically meaningful improvement in acne severity as measured by IGA scores (Figure 1, Panel A). At baseline, all patients presented with severe inflammatory acne, with IGA scores of 4 (n = 3) or 5 (n = 2). All patients demonstrated marked clinical improvement, with IGA scores improving by 3 to 4 points from baseline (individual results reported in Table 2). At the one-month follow-up visit, IGA scores were 0 (n = 3) or 1 (n = 2), corresponding to complete clearance or minimal residual acne activity in all cases. These improvements were maintained at the three-month follow-up, with an identical distribution of IGA scores, demonstrating sustained therapeutic effect (Figure 1, Panel A).

Baseline ECCA scores ranged from 225 to 340 points (median 295), reflecting a severe to very severe atrophic scarring burden across all patients. At the one-month follow-up, a marked reduction was observed, with scores decreasing to 0-95 (median = 60). Individual percentage reductions ranged from 66% to 100% compared to baseline, with two patients achieving complete ECCA clearance (score of 0). Further improvement was documented at the three-month follow-up, yielding a mean reduction of 87.9% from baseline (range = 77.4%-100%). Two patients maintained complete clearance at three months (Figure 1, Panel B).

Baseline Goodman & Baron scores ranged from 27 to 34 points, categorizing all patients within the severe scarring range. At the one‑month follow‑up, a clear reduction was observed, with scores decreasing to 0-11 (median = 9). Individual percentage reductions ranged from early partial improvement to complete clearance, with one patient achieving a score of 0 at one month. At the three‑month follow‑up, further improvement was documented, with a mean percentage reduction of 84.4% from baseline (range = 48.4%-100%). Complete clearance was observed in three patients (Figure 1, Panel C).

Notably, one patient demonstrated an initial improvement in the Goodman & Baron score from baseline (31) to one month (10, 68% reduction), but showed a subsequent increase at the three-month follow-up (16), resulting in a final reduction of 48.4% from baseline. All other patients maintained or continued to improve their scarring scores throughout the follow-up period.

**Figure 2:**
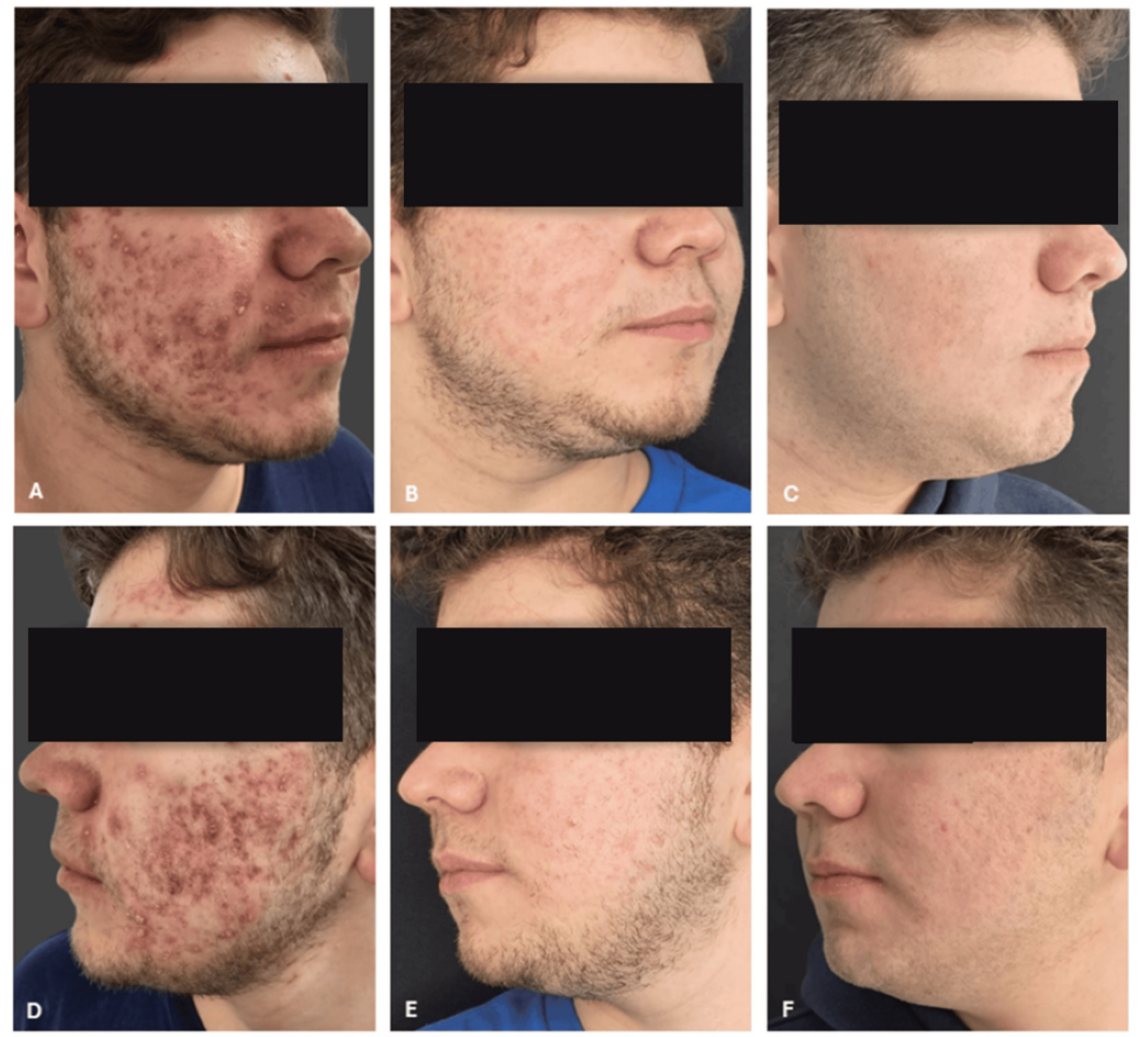
Sequential clinical improvement in a 20-year-old male with severe acne and atrophic scarring. The images illustrate the rapid therapeutic response to combined low-dose isotretinoin and multimodal laser therapy. (A-C) Left profile views and (D-F) right profile views at baseline, one-month follow-up, and three-month follow-up, respectively. At baseline (A, D), the patient presented with severe inflammatory acne (IGA, 5) and significant atrophic scarring (Goodman & Baron, 31; ECCA, 325). By the one-month follow-up (B, E), there was a nearly complete clearance of active inflammatory lesions (IGA, 1). At the final three-month follow-up (C, F), the patient demonstrated a marked quantitative reduction in atrophic scar severity, as assessed by the ECCA and Goodman & Baron scoring systems. IGA = Investigator’s Global Assessment; ECCA = Echelle D'Evaluation Clinique des Cicatrices D'Acne

**Figure 3:**
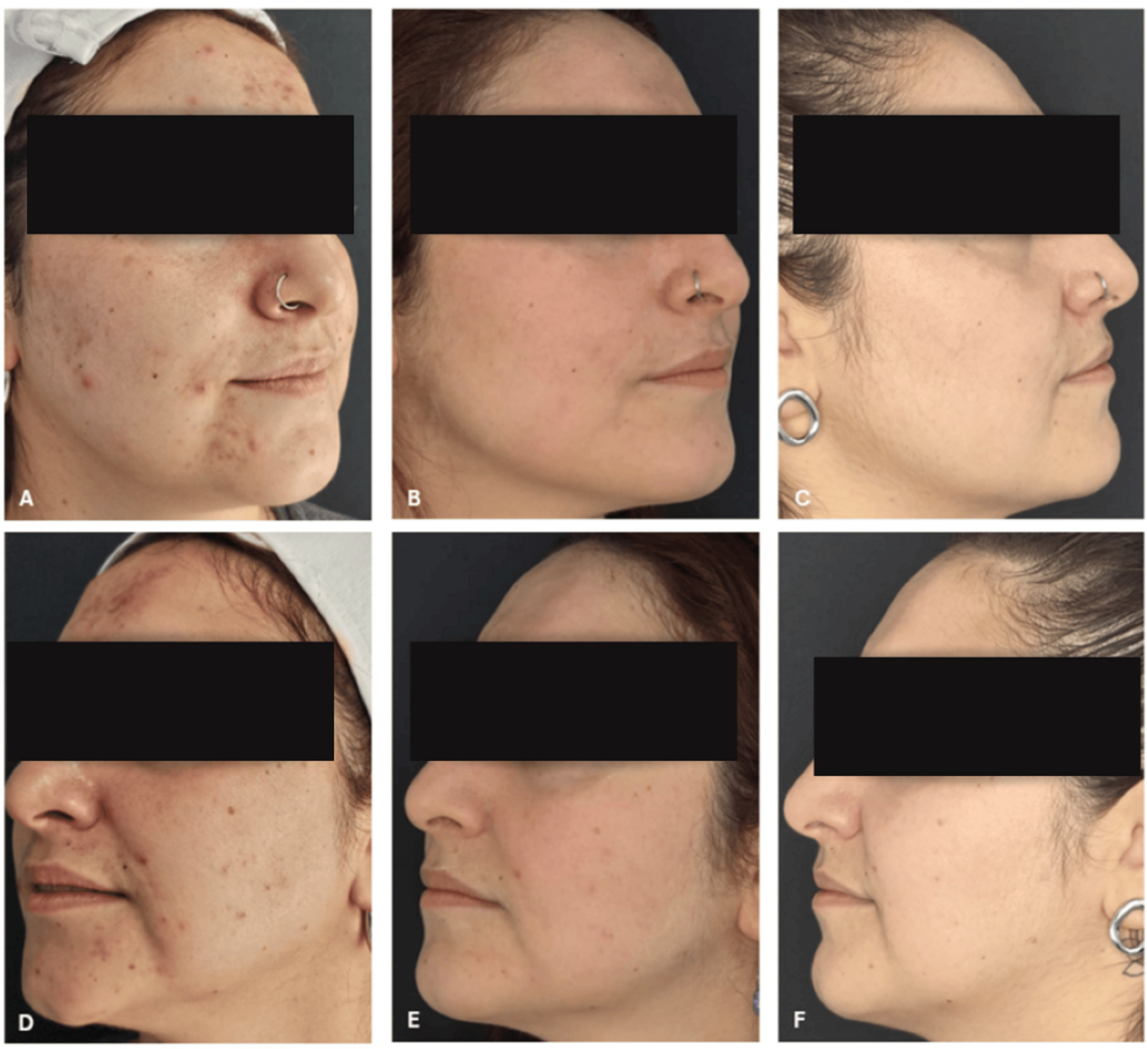
Sequential clinical improvement in a 29-year-old female with severe acne and atrophic scarring. The images illustrate the rapid therapeutic response to combined low-dose isotretinoin and multimodal laser therapy. (A-C) Left profile views and (D-F) right profile views at baseline, one-month follow-up, and three-month follow-up, respectively. At baseline (A, D), the patient presented with severe inflammatory acne (IGA, 4) and significant atrophic scarring (Goodman & Baron, 31; ECCA, 225). By the one-month follow-up (B, E), there was a complete clearance of active inflammatory lesions (IGA, 0). At the final three-month follow-up (C, F), the patient achieved complete resolution of atrophic scars, reaching a score of 0 on both the Goodman & Baron and ECCA scales. IGA = Investigator’s Global Assessment; ECCA = Echelle D'Evaluation Clinique des Cicatrices D'Acne

Representative clinical outcomes are illustrated in Figure 2 and Figure 3.

The combined treatment protocol demonstrated an excellent safety profile throughout the study period. No serious adverse events were reported in any patient. The most common side effect was mild transient erythema, which occurred in all patients following each laser session and resolved spontaneously within 48-72 hours without requiring any medical intervention or topical corticosteroids.

No cases of prolonged erythema (>7 days), post-inflammatory hyperpigmentation, post-inflammatory hypopigmentation, scarring, infection, blistering, or significant edema were observed during the treatment course or follow-up period.

Treatment tolerability was excellent, with pain scores consistently low throughout the protocol. All recorded VAS values ranged from 0 to 2 on a 0-10 scale, indicating minimal procedural discomfort. All patients reported being extremely satisfied with treatment outcomes at the final follow-up.

## Discussion

This case series study indicates that a combined protocol of low-dose systemic isotretinoin (20 mg/day) and sequential multimodal laser therapy, comprising 1,540 nm non-ablative fractional Er:Glass, fractional CO₂, and 1,064 nm Nd:YAG lasers, can be safely implemented and is associated with clinically meaningful improvements in patients presenting with simultaneous active inflammatory acne and atrophic scarring. To our knowledge, this is the first report to evaluate a triple-wavelength sequential laser regimen in this specific clinical context, providing preliminary evidence for the feasibility of integrated early intervention in one of dermatology’s most therapeutically challenging scenarios.

The magnitude and rapidity of acne improvement were noteworthy. Despite presenting with severe inflammatory disease (IGA 4-5 in all patients), all participants achieved near-complete or complete clearance (IGA 0-1) within one month of initiating the combined protocol, a response that was fully maintained at the three-month follow-up. While isotretinoin alone is well established as the most effective agent for severe inflammatory acne, capable of inducing prolonged remission through suppression of sebaceous gland activity, normalization of follicular keratinization, and reduction of inflammatory mediators, the speed and completeness of response observed here may reflect a synergistic contribution from the laser modalities, particularly the 1,064 nm Nd:YAG, whose thermal and photoacoustic effects on sebaceous glands may have accelerated clearance [23].

The improvements in atrophic scarring were no less significant. When contextualized against published monotherapy data, the magnitude of scar improvement observed in our case series appears notably higher. Fractional CO₂ and non-ablative fractional 1,540 nm lasers used alone have typically yielded ECCA reductions in the range of 14-59% and below 35%, respectively, while long-pulsed Nd:YAG 1,064 nm monotherapy has been associated with more limited gains, generally not exceeding 15-25% across commonly used scar scoring systems [24-32]. A comparable pattern emerges with Goodman & Baron scoring, where complete clearance with single-modality approaches remains uncommon [33,34]. In contrast, our sequential protocol achieved mean reductions of 87.9% and 84.4% on ECCA and Goodman & Baron scores, respectively, at the three-month follow-up, with complete clearance documented in a relevant proportion of patients.

While direct comparisons are limited by differences in study design, scar severity, and outcome measures, the magnitude of improvement observed in this case series supports the feasibility and potential clinical value of a sequential triple-wavelength approach delivered concurrently with low‑dose isotretinoin.

The rationale for combining three distinct wavelengths within a single session reflects the complementary biological mechanisms through which each modality acts on acne-affected and scarred skin.

The 1,540 nm non-ablative fractional erbium-doped glass laser and the fractional CO₂ laser both exert their effects primarily through controlled thermal injury, albeit at different tissue depths and with different degrees of epidermal disruption. While the CO₂ component provides micro-ablative remodeling of fibrotic scar tissue [35], the 1,540 nm wavelength, whose primary chromophore is water, with additional absorption by sebaceous glands, contributes to deep dermal coagulation without full epidermal ablation [36]. This thermal damage initiates the classical wound-healing cascade, inflammation, proliferation, and remodeling, with downstream upregulation of key molecular mediators, including transforming growth factor beta, matrix metalloproteinases, basic fibroblast growth factor, and heat shock proteins, ultimately driving collagen remodeling and dermal volume restoration [35].

The 1,064 nm Nd:YAG laser, by contrast, operates through fundamentally different mechanisms depending on the mode employed. In Q-switched mode, delivered through a fractional handpiece, the laser generates laser-induced optical breakdown (LIOB), producing intra-dermal vacuoles through a photoacoustic and photomechanical effect in the absence of thermal damage [37,38]. This mechanical stimulus is thought to directly activate fibroblasts via mechanotransduction pathways, promoting de novo synthesis of collagen and elastic fibers through cytokine signaling [39,40], a biological effect that is additive to, rather than redundant with, the thermally mediated cascade of the CO₂ and 1,540 nm components. Because this mechanism does not rely on epidermal disruption or thermal injury, re-epithelialization is not required, the risk window for post-inflammatory hyperpigmentation and infection is reduced, and treatment can be layered within the same session without compounding thermal load. Additionally, the Q-switched 1,064 nm laser may confer a secondary benefit in managing post-inflammatory hyperpigmentation, a frequent sequela of inflammatory acne, through its selective targeting of epidermal and dermal melanin.

In quasi-long-pulsed mode, the 1,064 nm laser serves an additional and distinct function: the targeted photocoagulation of superficial vascular structures [41]. Persistent erythematous macules, representing immature or inflamed vascular lesions within repairing scar tissue, are among the most common findings during active acne treatment and constitute a relevant therapeutic target. Because the chromophore for the 1,064 nm wavelength in this context is oxyhemoglobin, the laser selectively addresses the vascular component without significant epidermal impact, providing an anti-inflammatory effect and promoting vascular remodeling in a tissue compartment that neither the 1,540 nm nor the CO₂ laser targets effectively.

Taken together, this triple-wavelength approach integrates three biologically distinct mechanisms, thermal ablation and coagulation, photoacoustic mechanotransduction, and vascular photocoagulation, into a single treatment session, potentially achieving a quality and completeness of tissue remodeling that exceeds what any individual modality could produce alone.

The absence of wound healing complications, keloid formation, or abnormal scarring in this cohort, despite concurrent use of laser and isotretinoin, represents the most clinically relevant finding of this study, and aligns with the growing body of evidence supporting the safety of modern laser technologies in this setting [8]. The historical concerns underpinning the long-standing 6-12-month waiting period originated from case reports of hypertrophic scarring and delayed re-epithelialization following fully ablative procedures, mechanical dermabrasion and continuous-wave CO₂ resurfacing, performed during high-dose isotretinoin therapy. These early modalities caused extensive, confluent epidermal disruption with no adjacent reservoir of uninjured tissue to support re-epithelialization, a fundamentally different tissue injury profile from that of modern fractional platforms.

Contemporary fractional lasers create arrays of microscopic treatment zones surrounded by intact tissue, preserving a viable epidermal reservoir that enables rapid wound closure and substantially reduces the risk of delayed healing. The 1,540 nm non-ablative component preserves the epidermis entirely, while the fractional CO₂, though micro-ablative, limits direct thermal injury to a fraction of the treated surface. The progressive incorporation of the 1,064 nm Nd:YAG, a wavelength that does not ablate the epidermis, further reduced procedural risk while contributing anti-inflammatory and vascular remodeling effects. The low fixed isotretinoin dose employed (20 mg/day), well below the conventional therapeutic range, likely further mitigated any residual concern about impaired wound healing, while maintaining disease control through extended treatment duration.

One patient in the present series demonstrated a divergent trend between scar assessment scales during follow‑up, with an initial marked improvement in the Goodman & Baron score at one month, followed by a partial increase at three months, while the ECCA score continued to decrease over the same interval. This discrepancy underscores the different dimensions captured by the two grading systems. The Goodman & Baron quantitative scale places greater emphasis on individual scar morphology, surface texture, and visual perception, which may be influenced by transient factors such as residual erythema, localized inflammation, edema, or short‑term changes in scar palpability. In contrast, the ECCA score integrates scar type, extent, and anatomical distribution, providing a more global assessment of overall scarring burden. The continued reduction in ECCA score in this patient suggests ongoing global improvement in scar severity despite short‑term fluctuations in Goodman & Baron scoring. Importantly, this patient still exhibited a net reduction from baseline across both scales and reported high satisfaction with treatment outcomes. These findings highlight the dynamic and non‑linear nature of early scar remodeling and emphasize the value of using complementary scoring systems to capture different aspects of treatment response in the early post‑intervention period.

Limitations

This study has several limitations that must be acknowledged. The small sample size, retrospective design, and single-center setting limit generalizability and preclude any formal statistical analysis of outcomes. The absence of a control group makes it impossible to disentangle the respective contributions of isotretinoin and laser therapy to the improvements observed, or to directly compare the combined approach against either modality alone. In addition, outcome assessments were not blinded and were performed by the treating clinicians, which represents a methodological limitation inherent to uncontrolled case series; however, validated quantitative scoring systems were used to standardize evaluations and minimize subjective interpretation. The three-month post-treatment follow-up is insufficient to capture the full trajectory of collagen remodeling, which may continue for six months or beyond after fractional resurfacing, nor does it allow assessment of long-term durability or scar recurrence. Finally, the cohort comprised exclusively Fitzpatrick skin types II-IV; extrapolation to higher phototypes, which carry a greater risk of post-inflammatory pigmentary complications following ablative procedures, requires specific investigation.

## Conclusions

This case series provides preliminary evidence that low-dose systemic isotretinoin combined with sequential multimodal laser therapy (1,540 nm non‑ablative fractional, fractional CO₂, and 1,064 nm Nd:YAG) can be safely implemented and is associated with clinically meaningful improvements in patients with concurrent active inflammatory acne and severe atrophic scarring. Rapid and sustained acne clearance was achieved alongside substantial improvement in scar severity, without wound‑healing complications or pigmentary adverse events, despite the use of fractional ablative lasers during isotretinoin therapy. While these findings derive from a small uncontrolled cohort and should therefore be interpreted with appropriate caution, they challenge the long-standing paradigm of sequential management and support the feasibility of simultaneous early intervention, before irreversible dermal damage accumulates. Prospective controlled studies with larger cohorts and extended follow-up are warranted to confirm these observations and to assess their applicability across broader clinical settings.
